# Hope for Restoration of Dead Valuable Bulls through Cloning Using Donor Somatic Cells Isolated from Cryopreserved Semen

**DOI:** 10.1371/journal.pone.0090755

**Published:** 2014-03-10

**Authors:** Naresh L. Selokar, Monika Saini, Prabhat Palta, Manmohan S. Chauhan, Radheysham Manik, Suresh K. Singla

**Affiliations:** 1 Animal Biotechnology Centre, National Dairy Research Institute, Karnal, India; 2 Cellular Reprogramming Lab., Department of Animal Physiology and Reproduction, Central Institute for Research on Buffaloes, Hisar, India; Justus-Liebig-Universität, Germany

## Abstract

Somatic cells were isolated from cryopreserved semen of 4 buffalo bulls, 3 of which had died over 10 years earlier, and were established in culture. The cells expressed cytokeratin-18, keratin and vimentin indicating that they were of epithelial origin. The cells were used as nuclear donors for hand-made cloning for producing buffalo embryos. The blastocyst rate and quality, as indicated by apoptotic index, were comparable among embryos produced using cells obtained from fresh or frozen-thawed semen or those obtained from conventional cell sources such as skin. Examination of the epigenetic status revealed that the global level of H3K27me3 but not that of H3K9/14ac and H4K5ac differed significantly (P<0.05) among cloned embryos from different bulls. The relative mRNA abundance of *HDAC1*, *DNMT1*, *P53* and *CASPASE 3* but not that of *DNMT3a* differed in cells and in cloned embryos. Following transfer of 24 cloned embryos produced from fresh semen-derived cells to 12 recipients, one calf weighing 55 kg, which is now 6 months of age and is normal, was born through normal parturition. Following transfer of 20 embryos produced from frozen-thawed semen-derived cells to 10 recipients, 2 became pregnant, one of which aborted in the first trimester; the calf born was severely underweight (17 kg), and died 12 h after birth. The ability of cells derived from fresh and frozen-thawed semen to produce live offspring confirms the ability of these cells to be reprogrammed. Our findings pave the way for restoration of highly precious progeny-tested bulls, which has immense economic importance, and can also be used for restoration of endangered species.

## Introduction

Restoration of a dead individual has always been a fascinating issue. Unlike wild animals, for which getting viable genetic material is a major hurdle in restoring them, genetic material may be available in the form of cryopreserved semen in case of farm animals. Although the functional integrity of somatic cells is lost if frozen without efficient cryopreservation, genome remains intact in 60% of somatic cells even after lyophilization, and lyophilized nuclei injected into enucleated oocytes can develop to normal cloned embryos following somatic cell nuclear transfer (SCNT) [1]. SCNT, which has been successfully applied to produce endangered [2]–[4] and exotic [5] animals holds a lot of potential for preservation or restoration of endangered, exotic, or even extinct animal species if somatic cells of such animals are available. Cryopreservation of semen allows preservation of somatic cells, which can be isolated, cultured and proliferated for use in production of progeny through SCNT. This approach could enable restoration of valuable high genetic merit progeny-tested bulls which may be dead but the cryopreserved semen of which is available. This holds considerable economic promise through dissemination of semen of these bulls. This approach would also enable reintroduction of previously inaccessible genes from earlier generations. Banks of cryopreserved semen could therefore serve as a potential source of novel genes that could be reincorporated into an animal population with a limited gene pool. Previously, somatic cells isolated from fresh semen have been successfully used for the production of cloned bovine embryos although attempts to produce cloned embryos from cryopreserved semen were so far unsuccessful [6]. In the present study, we succeeded in isolation and establishment of somatic cells from frozen-thawed semen stored for over a decade, production of cloned embryos from these cells and establishing pregnancies from these embryos leading to birth of live offspring following their transfer to recipients.

Following SCNT, correct acetylation and methylation of nuclear histones is necessary for efficient reprogramming of differentiated somatic cells into a totipotent state [7]. There is evidence that reprogramming potential of donor cells is dependent on their epigenetic state [8]. Considering the importance of the epigenetic status of the donor cells, we studied the epigenetic status and expression of some epigenetically important genes in semen- and skin-derived cells and in cloned embryos produced using these cells in the present study. Since the ultimate capability of any type of donor cell to produce cloned embryos is established only after production of live offspring, we also studied in vivo developmental potential of cloned embryos produced from both frozen-thawed and fresh semen-derived somatic cells.

## Materials and Methods

In vitro culture of somatic cells, oocytes and embryos was done at 38.5°C in a CO_2_ incubator (5% CO_2_ in air). Animal experiments were carried out after approval by Committee for the Purpose of Control and Supervision on Experiments on Animals (Indian Council of Medical Research, New Delhi) and the Animal Ethics Committee (National Dairy Research Institute, Karnal).

### Isolation and culture of ear-derived somatic cells

Buffalo ear skin cells were isolated and cultured. Briefly, ear skin tissue from buffalo was aseptically collected with the help of an ear notcher in sterile Ca^2+^and Mg^2+^-containing Dulbecco's phosphate buffered saline (DPBS) supplemented with 50 µg/ml gentamicin sulfate. The tissue was finely cut into 1–2 mm size pieces, which were cultured in 10 µl of DMEM/F12 medium (1∶1 ratio), supplemented with 20% FBS, 0.68 mM L-glutamine and 50 µg/ml gentamicin sulfate in a CO_2_ incubator in T-25 culture flasks. After 12 h, 3 ml of fresh medium was added to the flask to keep the attached explants submerged. The cells from the outgrowth were removed by trypsinization after they reached confluence, which usually took 5–7 days. The cells were subcultured and grown in T-25 flasks till they attained confluence following which they were passaged up to 10 times. Aliquots of cells at early passages (passage 2–3) were cryopreserved in DMEM/F12 containing 10% dimethyl sulphoxide (DMSO) and 20% FBS, and were stored in liquid nitrogen for future use.

### Isolation and culture of semen-derived somatic cells

Two methods were followed for the isolation of semen-derived somatic cells. In the first method, fresh semen, diluted 1∶5 with DPBS was centrifuged at 400× g for 10 min, and the pellet was washed 3 times with DPBS. The pellet was resuspended in 500 µl of DMEM/F12 medium and the contents were cultured in 4-well dish in DMEM/F-12 supplemented with 20% FBS, 10 ng/ml epidermal growth factor (EGF), 5 µg/ml insulin, 0.5 µg/ml hydrocortisone, 200 µg/ml gentamycin sulfate, 25 µg/ml amphotericin B, 100 IU/ml penicillin and 0.1 mg/ml streptomycin.

In the second method, somatic cells were isolated using a procedure described earlier with modifications [9]. Briefly, fresh semen was diluted 1∶5 in DPBS and 2.5 ml of it was layered over a column of 20, 50, and 90% Percoll (2.5 ml each) in a 15 ml Falcon tube. For the isolation of somatic cells from frozen-thawed semen, the contents of 8–10 straws were pooled and layered over Percoll in a 15 ml Falcon tube as described above for fresh semen. The Falcon tube was centrifuged at 400× g for 25 min after which contents of the 20% layer were collected and were washed 2–3 times with DPBS by centrifugation at 400× g for 10 min. The contents were then washed once with DMEM/F12 supplemented with 10% FBS, and were cultured in 4-well dish in the culture medium used in the first method. The medium was changed with fresh medium after 48 h to remove the unattached cells and spermatozoa. The concentration of gentamycin sulfate in the medium was reduced stepwise to 150, 100 and finally to 50 µg/ml within a week, and amphotericin B was excluded from the culture medium after 1–2 weeks. The cells were cultured in the culture medium (DMEM/F-12+10% FBS+10 ng/ml EGF+5 µg/ml insulin+0.5 µg/ml hydrocortisone+50 µg/ml gentamycin sulfate) until confluence, which usually took 20–30 days, with a change of medium twice a week. The cells were then passaged up to 10 times. The attachment and proliferation rate of the cells was assessed for each sample. Some of the aliquots were cryopreserved and others were kept in culture to optimize the culture conditions and to examine the growth characteristic and the epigenetic status to investigate their potential as donor cells for production of cloned embryos. Donor cells between passages 5 to 10 were used in different experiments, including those on SCNT, to avoid differences caused by the ageing of cells.

Aliquots of cells (approximately 1×10^5^ cells/ml) at early passages (passage 2–3) were subjected to slow freezing at the rate of 1.0°C/min to −80°C in DMEM/F12 containing 10% dimethyl sulphoxide (DMSO) and 20% FBS in Cryovials, after which the Cryovials were stored in liquid nitrogen. Whenever required, the cells were thawed and washed once with the culture medium before being seeded.

### Immunocytochemical characterization of cells

Cells at passage 2–3 were cultured in 96-well plates (approximately 2000 cells/well) until they attained 70–80% confluence following which they were fixed for 1 h in 4% paraformaldehyde (in DPBS). The cells were permeabilized by treatment with 0.5% Triton X-100 for 30 min, blocked with 3% BSA, incubated for 1 h with the primary antibody (mouse anti-cytokeratin, 1∶500, SC-32329, Santa Cruz Biotechnology; anti-keratin, 1∶500, MAB1611, Millipore; mouse anti-vimentin 1∶500; V6630, Sigma) diluted in the blocking solution, and then with the secondary antibody (goat anti-mouse/rabbit IgG) conjugated with fluorescein isothiocyanate (FITC) for 1 h. Positive controls used for testing the staining protocol were from each respective cell culture and were labeled with mouse anti-tubulin (1∶500, T8328, Sigma) whereas the addition of the primary antibody was omitted in the negative controls. The cells were incubated for 10 min in10 µg/ml Hoechst 33342 for staining the nuclei. For detecting the fluorescence, the cells were examined by epifluorescence microscopy. The images were merged using Adobe Photoshop CS 8.0.1 software (Adobe Systems Inc., San Jose, CA, USA).

### Examination of morphology and growth characteristic of cells

Morphological changes in the cells were examined by taking images on an inverted microscope (Nikon, Tokyo, Japan) at 200× magnifications. The population doubling time was determined using the software (http://www.doubling-time.com) after the cells had been cultured for 72 h. For examining the cell proliferation, the cells were cultured for 120 h after which the number of cells/well was counted using a TC20™ automated cell counter (Bio-Rad Laboratories, Hercules, CA) every 24 h after trypsinization. The medium was changed with fresh medium at 72 h.

### Production of cloned embryos and assessment of fusion efficiency, embryo development and apoptosis

Somatic cells were synchronized in G1 stage of cell cycle by growing them in culture to full confluence for contact inhibition following which they were used as donor nuclei by making single cell suspensions as described previously [10]. Preparation of recipient oocytes (in vitro maturation, cumulus/zona removal and manual enucleation), fusion, activation and in vitro culture (IVC) of cloned embryos were performed according to the methods described earlier [11].

Following examination of the fusion efficiency 30 min after reconstruction, the unfused/lysed embryos were discarded and the remaining intact fused embryos were subjected to activation. The cleavage and blastocyst rate was recorded on day 8 of IVC. For assessment of embryo quality, total cell number (TCN) and the level of apoptosis in day 8 blastocysts were determined by TUNEL staining. Briefly, the blastocysts were washed 3 times with Ca^2+^- and Mg^2+^-free DPBS containing 0.3% polyvinyl alcohol (PVA) in 4-well dish and were fixed in 4% paraformaldehyde for 1 h at 37°C. These were then washed thrice with Ca^2+^- and Mg^2+^-free DPBS containing 0.3% PVA and were kept at 4°C until the start of staining. The blastocysts were permeabilized by incubation with 0.5% Triton X-100 for 40 min after which these were incubated with FITC-conjugated dUTP and terminal deoxynucleotidyl transferase (TdT) for 1 h at 37°C in dark. The blastocysts were then incubated with the nuclear staining solution (10 µg/ml propidium iodide and 50 µg/ml RNase in Ca^2+^- and Mg^2+^-free DPBS containing 0.3% PVA) for 20 min at 37°C. In the negative controls, only FITC-conjugated dUTP was used whereas in the positive controls, the blastocysts were incubated with DNase solution (100 U/mL) for 20 min at 37°C prior to incubation with FITC-conjugated dUTP and TdT. The stained blastocysts were washed and mounted on glass slides in 3 µl droplets of antifade solution and were flattened with a cover slip. The images were captured at both red and green filters for examining the nuclei and the site of apoptosis, respectively. The merged images generated from red and green filters showing yellow bodies on the exact site of red nuclei were considered as apoptotic cells. Cell counting was performed from digital images obtained via an inverted Nikon fluorescence microscope. The total apoptotic indices were calculated for each embryo as follows: Apoptotic index in the blastocyst = (number of TUNEL-positive nuclei/total number of nuclei in blastocyst)×100.

### Cryosurvival of cloned blastocysts following vitrification in Open-pulled straws (OPS)

OPS vitrification was performed as described earlier [12], [13]. Briefly, cloned embryos which appeared to be morphologically normal and which had a distinct inner cell mass (ICM) were first transferred to T20 (TCM 199+20% FBS) after which they were incubated for 5 min at 38.5°C in the equilibration solution comprising 8.5% ethylene glycol (EG) and 8.5% DMSO in T20 medium. After an initial shrinkage and recovery, they were placed in the vitrification solution (16.5% EG, 16.5% DMSO, 0.5 M sucrose in T20) for 35–40 sec and were then immediately loaded in OPS by capillary action. The OPS were plunged in liquid nitrogen within 1 min of exposure to the vitrification solution. For thawing, the OPS were immersed directly in 1 M sucrose in T20. The blastocysts were equilibrated in this solution for 1 min and were then incubated in 0.5 M and 0.25 M sucrose in T20 for 5 min each. Subsequently, the blastocysts were incubated for 10 min in T20 without sucrose. The survival rate of blastocysts subjected to vitrification in OPS was determined by examination of their re-expansion after 22–24 h of culture in a CO_2_ incubator at 38.5°C in 4-well dish in RVCL medium (K-RVCL50, Cook, Australia).

### Global DNA methylation analysis

An ELISA-based kit (MDQ1, Imprint® Methylated DNA Quantification Kit, Sigma) was used as per the manufacturer's protocol to determine the global level of DNA methylation in somatic cells derived from Mu-5926 and Mu-4393. Briefly, genomic DNA, isolated from cells according to the method provided in a commercially available kit (NucleoSpin® Tissue XS, Macherey-Nagel Neumann-Neander-Straße 6–8 52355 Düren, Germany), was diluted to 100 ng/µl. DNA (100 ng in 50 µl of binding buffer) was then loaded into each well of the 8-well strip, and was incubated for 60 min at 37°C. The samples were blocked with 150 µl blocking solution, and were incubated with the capture (1∶1200) and detection (1∶1200) antibody for 60 and 30 min, respectively. After that, 100 µl of the developing solution was added to each well, leading to development of blue color, which turns to yellow after addition of the stop solution. The absorbance was read at 450 nm immediately. Quantification of global DNA methylation was carried out by calculating the amount of methylated DNA in the sample relative to global DNA methylated in a positive methylated control.

### Immunofluorescence staining for epigenetic markers in somatic cells and embryos

Global H3K9/14ac, H4K5ac and H3K27me3 analysis in donor cells and blastocysts was done by immunofluorescence staining. Briefly, somatic cells and cloned blastocysts were fixed with 4% paraformaldehyde, washed 3 times with Ca^2+^- and Mg^2+^-free DPBS containing 0.3% PVA, permeabilized with 0.5% Triton X-100 and then blocked with 3% BSA. The cells were then incubated overnight at 4°C with the respective primary antibody (anti-H3K9/14ac, 1∶1000, Santa Cruz Biotechnology, CA, USA; anti-H4K5ac, 1∶1500, Millipore, MA, USA; anti-H3K27me3, 1∶1500, Millipore) diluted in 3% BSA. After washing 5 times with Ca^2+^- and Mg^2+^-free DPBS containing 0.3% PVA and 0.1% Triton X-100, the cells/blastocysts were incubated for 90 min with FITC-conjugated goat anti-rabbit secondary antibody (Sigma) diluted 1∶700 in Ca^2+^- and Mg^2+^-free DPBS containing 0.3% PVA. After washing 5 times with Ca^2+^- and Mg^2+^-free DPBS containing 0.3% PVA and 0.1% Triton X-100, the nuclei were counterstained with Hoechst 33342 (10 µg/ml) and rinsed with Ca^2+^- and Mg^2+^-free DPBS containing 0.3% PVA and 0.1% Triton X-100. The stained cells/embryos were mounted on slides in the mounting medium (2.5% DABCO, Sigma, in glycerol), and were observed under a Nikon fluorescence microscope. NIS-element basic research image processing software (Nikon) equipped with the microscope was used for image acquisition and quantitative measurements of the mean pixel intensity emitted by each individual nucleus. At least 10 images of somatic cells (200 nuclei from each image) or 10 blastocysts (∼75 nuclei/blastocyst) were analyzed for each epigenetic marker.

### Gene expression analysis in donor cells and embryos

RNA was isolated from cells (1×10^4^) or blastocysts (n = 3 to 4) using RNAqueous micro kit (Ambion, Austin, TX, USA) according to the manufacturer's instructions. The genomic DNA contamination was removed by DNase treatment at 37°C for 20 min. The RT reaction was carried out using the M-MLV RT provided in superscript reverse transcriptase III kit (Invitrogen). Quantitative real-time PCR (qPCR) was performed using the optimized primer sets shown in Table S1 on a CFX96 real-time system (Bio-Rad) with maxima@SYBR Green master mix (Fermentas, St. Leon-Rot, Germany) at the following thermal cycling conditions: 95°C for 5 min, followed by 40 PCR cycles of 95°C for 15 sec, 58°C for 30 sec, and 72°C for 30 sec. Melting peaks were determined by melting curve analysis in order to ensure specific amplification. Agarose (2%) gel electrophoresis analysis was carried out to determine the length of the amplified PCR products. Relative quantification of gene expression was conducted using a method described previously [14]. β-actin mRNA was employed as an internal standard for the analysis of relative transcript levels of each gene whereas in the negative controls, H_2_O replaced cDNA in the reaction tubes. For comparison, the average expression level of each gene from skin-derived cells or embryos was set as 1. Three separate experiments were performed with three replicates for each gene.

### Embryo transfer and detection of pregnancy

Cycling buffaloes possessing a functional corpus luteum were treated with PGF_2α_ analogue (Cloprostenol sodium, 500 mg) intramuscularly. Those exhibiting estrus 72 h after the treatment were selected as recipients. Two, day 7/8 blastocysts produced using donor cells obtained from fresh or frozen-thawed semen were transferred to each recipient, one each to the ipsilateral and contralateral uterine horn. Pregnancies were confirmed by ultrasonography at days 40–45 and were reconfirmed at day 60 by transrectal palpation.

### Confirmation of origin of frozen-thawed semen-derived somatic cells and cloned calves

Due to the presence of egg yolk in semen freezing medium, the individual and species identity of the cells isolated from cryopreserved semen was genetically confirmed with microsatellites markers analysis. To determine the origin of the established semen-derived cells, DNA was extracted from a frozen-thawed semen sample and from the cultured cells by phenol chloroform method, and was processed for microsatellite markers analysis. Also, microsatellite analysis was carried out to confirm the origin of the born cloned calves.

### Experimental design and statistical analysis

In experiment 1, the efficacy of simple centrifugation was compared with that of Percoll gradient centrifugation for the isolation of somatic cells from fresh semen. The parameters used were cell attachment and proliferation rate. In experiment 2, Percoll gradient centrifugation was used for the isolation of somatic cells from frozen-thawed semen samples, and the incidence of cell attachment and proliferation was compared among semen samples from 4 individual bulls. In experiment 3, the cultured somatic cells were characterized by examining their morphology and expression of cell type-specific markers (cytokeratin-18, keratin and vimentin) by immunofluorescence staining. In experiment 4, the cells were cultured in 6-well dish for up to 120 h. The population doubling time were examined at 72 h whereas the proliferation rate was determined up to 120 h. In experiment 5, to compare the efficacy of different attachment factors, the cells were cultured in 24-well dish coated with bovine collagen IV or laminin or with no attachment factor (controls) for 120 h. The cells were harvested every 24 h for determination of cell concentration. In experiment 6, cloned embryos were produced using skin- and semen-derived donor cells. The developmental competence was compared by recording the fusion, cleavage and blastocyst rate, and the embryo quality was compared by determining the TCN and the apoptotic index by TUNEL assay. In experiment 7, the cryosurvival rate of cloned blastocysts produced using skin- and semen-derived donor cells was compared by determining their re-expansion rate following vitrification in OPS. In experiment 8, DNA methylation and the global level of H3K9/14ac, H4K5ac, H3K27me3 and H3K18ac was determined in donor cells, and that of the first three markers was determined in cloned embryos by immunofluorescence staining whereas in experiment 9, the relative mRNA abundance of *HDAC1*, *DNMT1*, *DNMT3a*, *P53* and *CASPASE 3* was determined in donor cells and cloned embryos by qPCR. The in vivo developmental potential of cloned embryos produced from fresh/frozen-thawed semen-derived donor cells was determined by transferring them to recipient buffaloes. The genetic make-up of the cloned calves born and of the donor cells used for producing these calves was matched by microsatellite marker analysis.

Statistical analysis was performed using SYSTAT 12 (Systat Software, Inc. Chicago, IL, USA) software. Percentage values were analyzed after arcsine transformation. One-way ANOVA, together with Fisher's LSD was used to compare the means of different groups. The differences were considered to be significant at p<0.05.

## Results

### Isolation, culture and characterization of semen-derived somatic cells

In experiment 1, when the pellet obtained after centrifugation of fresh semen, which was expected to contain the somatic cells, motile and non-motile spermatozoa and debris, was resuspended in the culture medium and cultured in 4-well dish, no cell attachment was observed at day 3 in any of the 8 trials conducted. However, after Percoll gradient centrifugation, somatic cells, spermatozoa and debris were separated among different layers. A majority of somatic cells and non-motile sperm were obtained in the 20% layer, most of motile sperm and a few somatic cells were obtained in the 50% layer whereas the 90% layer was found to contain debris (Figure S1a). Following seeding, cell attachment was observed 5/8 (62.5%) times, and the attached cells proliferated during culture 4/5 (80%) times. The morphological appearance of semen-derived cells at different days of culture is presented in Figure S1b. In experiment 2, somatic cells were isolated by Percoll gradient centrifugation from semen samples from 4 individual bulls, 3 out of which had been cryopreserved in liquid nitrogen for over 10 years; the only bull alive (Mu-5926) was used for isolation of somatic cells from skin and from fresh and cryopreserved semen. Following seeding of somatic cells obtained from Mu-5926, Mu-4393 and Mu-3567, the frequency of cell attachment was 4/4 (100%), 3/5 (60%) and 3/3 (100%), respectively, whereas the frequency of cell proliferation was 2/4 (50%), 1/3 (33.3%) and 3/3 (100%), respectively. No cell proliferation was observed for semen sample of Mu-4371 although cell attachment was observed at a frequency of 3/5 (60%). In experiment 3, ear skin-derived cells were found to express vimentin but not keratin and cytokeratin-18 indicating that they were of fibroblast origin. Somatic cells isolated from fresh and cryopreserved semen had classic cobblestone morphology (Figure S2a), and they expressed both cytokeratin-18 and keratin (Figure S2b), indicating that they were of epithelial origin. These cells also expressed vimentin. Since the diluents used for dilution of semen before freezing sometimes contain milk whey or egg yolk, which may contain somatic cells, we confirmed by microsatellites analysis that the established somatic cells were from the cryopreserved semen of the respective bull (Table S2).

### Growth characteristic of cells

In experiment 4, a comparison of the of skin-, fresh semen- and frozen-thawed semen-derived somatic cells from Mu-5926 revealed that the population doubling time of skin-derived cells was lower (P<0.05) than that of the cells derived from fresh or frozen-thawed semen (Figure S2c). Among the three bulls, the population doubling time was significantly different (P<0.05) in the order Mu-3567> Mu-5926> Mu-4393. Therefore, somatic cells from the semen of Mu-4393 were selected for experiment 5 for comparison of the efficacy of different attachment factors. It was found that whereas for the skin-derived cells, the cell proliferation rate was similar at 24, 48, 72, 96 and 120 h of culture, for the semen-derived cells, the cell proliferation was higher (P<0.05) in the order bovine collagen IV>laminin>controls at 72, 96 and 120 h of culture (Figure S2d,e).

Somatic cells isolated from frozen-thawed semen of Mu-3567 stopped dividing after 7–8 passages even though growth factors and collagen IV-coated surface were provided whereas cells isolated from both fresh and frozen-thawed semen of Mu-5926 and Mu-4393 were subcultured more than 15 times. Among the cells derived from frozen-thawed semen, the diameter of cells was different (P<0.05) in the order Mu-3567>Mu-5926>Mu-4393. The diameter of cells isolated from fresh and frozen-thawed semen was higher (P<0.05) than that of skin-derived cells in the case of Mu-5926 (Figure S2f). Following thawing, the survival rate of cells that had been subjected to slow freezing was similar for skin- and semen-derived cells after trypan blue staining (Figure S2g).

### Developmental competence and quality of cloned embryos

In experiment 6, the fusion efficiency was lower (P<0.05) for the cells derived from frozen-thawed semen of Mu-3567 (87±0.9%), compared to that of 100% for other groups (Table 1). The cleavage rate was lower (P<0.05) with the somatic cells derived from frozen-thawed semen compared to that with the skin-derived cells or those derived from fresh semen. However, the blastocyst rate of the three types of cells from Mu-5926 or those derived from frozen-thawed semen of Mu-4393 was not significantly different but was higher (P<0.05) than that obtained with cells from frozen-thawed semen of Mu-3567. Similarly, although the TCN was similar among all the groups, the apoptotic index was higher (P<0.05) for cloned embryos produced using cells derived from frozen-thawed semen of Mu-3567. In experiment 7, the cryosurvival rate of cloned embryos produced using skin- and semen-derived donor cells was similar as indicated by non-significant differences in their re-expansion rate following vitrification.

**Table 1:** Developmental competence, quality and cryosurvival rate of cloned embryos produced using different types of donor cells.

Animal number	Origin of donor cells	Embryos reconstructed (n)	Fused n (%)	Cleaved n (%)	Blastocysts n (%)	Total cell number	Apoptotic index	Blastocysts vitrified (n)	Blastocysts re-expanded after warming n (%)
Mu-5926	Skin	183	183 (100%)a	181 (99.0±0.65)a	86 (46.9±3.9)a	281.7±29.2	5.1±0.9a	27	13 (47.5±5.8)
	Fresh semen	183	183 (100%)a	180 (98.6±1.3)a	88 (48.8±4.4)a	299.3±30.3	5.3±0.8a	33	21 (63.8±2.8)
	Frozen-thawed semen	168	168 (100%)a	159 (94.4±2.0)b	84 (48.7± 4.5)a	278.7±20.3	5.4±0.7a	34	21 (62.1±2.7)
Mu- 4393	Frozen-thawed semen	256	256 (100%)a	237 (92.3±3.0)b	133 (51.4±3.3)a	272.5±68.4	5.7±0.6a	50	29 (55.7±4.9)
Mu-3567	Frozen-thawed semen	245	213 (87.0±0.9)b	182 (92.5±1.0)b	68 (34.3±3.0)b	223.0±38.2	20.1±4.2b	20	10 (51.5±15.1)

Data from 12 trials, Values with different letters within the same column differ significantly (P<0.05).

### Epigenetic status of donor cells and cloned embryos

In experiment 8, as indicated by mean pixel intensity after immunofluorescence staining, the global level of H3K9/14ac and H3K18ac was similar among donor cells derived from skin or fresh or frozen-thawed semen of Mu-5926 or those derived from frozen-thawed semen of Mu-4393. However, the level of H4K5ac and H3K27me3 was lower (P<0.05) in cells derived from the skin of Mu-5926 or those derived from frozen-thawed semen of Mu-4393 compared to that in cells derived from fresh or frozen-thawed semen of Mu-5926 (Figure S3).

**Figure 1:**
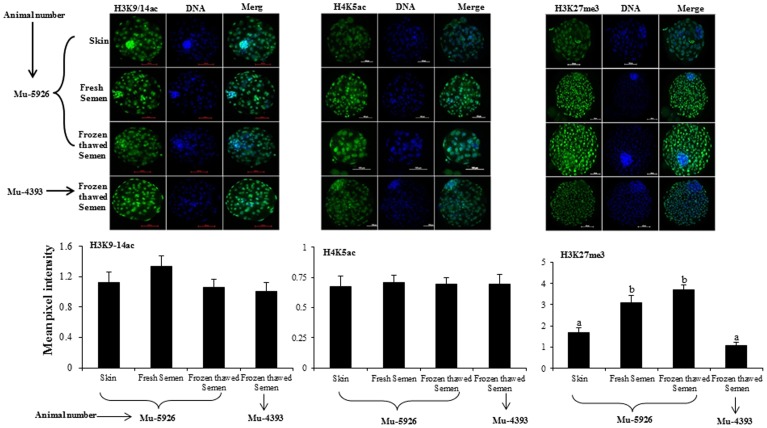
Mean pixel intensity of H3K9/14ac, H4K5ac and H3K27me3 examined by immunofluorescence staining in cloned blastocysts produced using cells derived from skin or fresh or frozen-thawed semen.

The global level of H3K9/14ac and H4K5ac was similar among cloned embryos produced using donor cells derived from skin or fresh or frozen-thawed semen of Mu-5926 or those derived from frozen-thawed semen of Mu-4393. However, the level of H3K27me3 was lower (P<0.05) in cloned embryos produced using donor cells derived from the skin of Mu-5926 or those derived from frozen-thawed semen of Mu-4393 compared to that in cloned embryos produced using cells derived from fresh or frozen-thawed semen of Mu-5926 (Figure 1).

### Gene expression analysis in donor cells and cloned embryos and DNA methylation in donor cell

**Figure 2:**
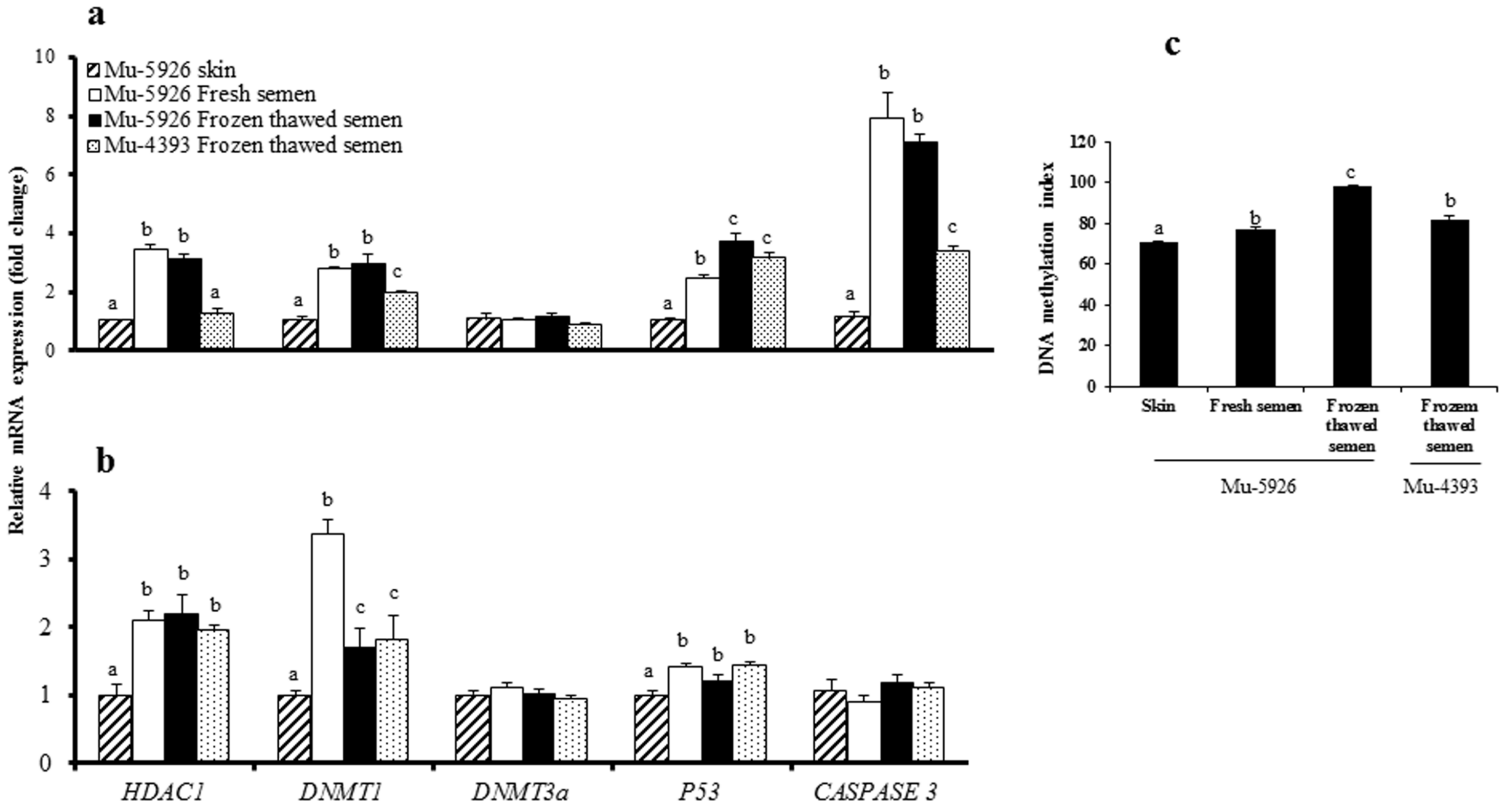
Relative mRNA abundance of some important genes in (a) cells derived from skin or fresh or frozen-thawed semen and in (b) cloned blastocysts produced using these cells, and (c) global level of DNA methylation in skin- and semen-derived cells.

In experiment 9, the relative mRNA abundance of *HDAC1*, *DNMT1*, *P53* and *CASPASE 3* was higher (P<0.05) in the donor cells obtained from fresh or frozen-thawed semen than that in skin-derived cells; this pattern was reflected in cloned embryos produced using these cells, except in case of *CASPASE 3*, the expression level of which was similar among the cloned embryos produced using the three types of cells (Figure 2a, b). The relative mRNA abundance of *DNMT3a* was similar among the three cell types and among the cloned embryos produced using these cells. The global level of DNA methylation was higher (P<0.05) in cells isolated from frozen-thawed semen than in those isolated from fresh semen which, in turn, was higher (P<0.05) than that in skin-derived cells (Figure 2c).

### Embryo transfer

**Figure 3:**
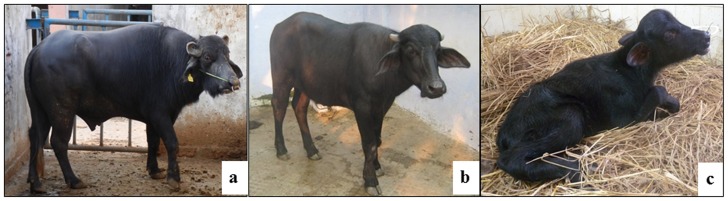
Cloned calves born. (a) Bull, the fresh semen of which was used; (b) cloned calf produced from fresh-semen-derived somatic cells at 6 months of age; (c) calf produced from transfer of frozen-thawed semen derived cloned embryos, which died after 12 h.

Following transfer of cloned embryos produced from fresh semen-derived cells, one each to the ipsilateral and contralateral uterine horn of twelve recipients, one recipient was pregnant, and it gave birth to an offspring weighing 55 kg through normal parturition. The calf is now 6 months of age and is normal (Figure 3). Following transfer of 20 embryos produced from frozen-thawed semen-derived cells to ten recipients in a similar manner, two were pregnant. One pregnancy aborted in the first trimester and one calf was born weighing 17 kg, which died 12 h after birth (Figure 3). DNA microsatellites analysis indicated that the cloned calves were derived from the respective cultured cells used as donors for SCNT (Table S3, S4).

## Discussion

To our knowledge, this is the first report on the production of live offspring from cells obtained from frozen-thawed or fresh semen through SCNT. The results of this study demonstrate that somatic cells can be isolated from frozen-thawed semen, established in culture and used as nuclear donors for producing cloned embryos with yield, quality, epigenetic status and reprogramming potential comparable to that of embryos produced using cells obtained from fresh semen or those obtained from conventional cell sources such as skin.

In our study, no cell attachment was observed following seeding of cells obtained after centrifugation of fresh semen. However, seeding of cells obtained from the 20% layer following Percoll gradient centrifugation led to their attachment and proliferation 5/8 (62.5%) and 4/5 (80%) times, respectively. This technique enabled isolation of somatic cells from cryopreserved semen that had been frozen over 10 years earlier. Following seeding, cell attachment and proliferation were observed in all frozen-thawed semen samples, the frequency of which varied between 60–100% and 0–100%, respectively, among individual bulls. The attempts to isolate culture and establish somatic cells from frozen-thawed semen have so far been unsuccessful [15]–[16]. One of the reasons for lack of success in establishing semen-derived cells in earlier studies could be the epithelial nature of these cells since, compared to fibroblasts, epithelial cells are more difficult to maintain in culture and require a more complex culture system [17]. It is also possible that the cryosurvival of the somatic cells used in our study may be different from that of cells used in earlier studies due to species differences and differences in cryoprotectants and protocols used. Most importantly, our semen dilution-rates never crossed 1∶20 for buffalo-bulls, whereas in most developed world, it is always 1∶60 or more. The lower dilution rates might have helped us in getting successful cell cultures.

Epithelial cells require specific culture conditions for optimum proliferation. Following a comparison of the proliferation rate of cells grown on bovine collagen IV, laminin or uncoated surface (controls) it was found that whereas the cell proliferation rate was not significantly different for the skin-derived cells, it was higher (P<0.05) in the order bovine collagen IV>laminin>controls for the semen-derived cells. In a similar study, proliferation of somatic cells obtained from fresh semen was greater when cells were cultured on the Matrigel-coated compared to collagen I-coated and uncoated plastic surface in sheep [15]. These results suggest that a suitable attachment factor may be necessary for optimal attachment and proliferation of semen-derived cells.

It appears that there may be substantial bull-to-bull variation in the quality of somatic cells isolated from frozen-thawed semen because cells obtained from Mu-3567 stopped dividing after 7–8 passages whereas those obtained from other bulls could be subcultured >15 times despite similar culture conditions. Also, the population doubling time and the diameter of cells were different (P<0.05) in the order Mu-3567>Mu-5926>Mu-4393, indicating that these could be inversely related. Cells from Mu-3567 not only had the lowest growth rate and the biggest size, but also the fusion efficiency and the blastocyst rate obtained with them was lower (P<0.05) and the apoptotic index was higher (P<0.05) than that for other bulls. Despite a small population size, it is tempting to speculate that the growth rate may be inversely related to the size. However, these trends need to be confirmed on a larger population of bulls. A comparison of the growth rate of three different types of cells obtained from the same animal revealed that the population doubling time of skin-derived cells was lower (P<0.05) than that of the cells derived from fresh or frozen-thawed semen.

In vitro embryo production efficiency is dependent upon the intrinsic developmental potency of the donor cell [16]. In our case, despite a lower (P<0.05) cleavage rate, the blastocyst rate was not significantly different between the cloned embryos produced from cells obtained from frozen-thawed semen and those produced using skin- or fresh-semen-derived cells. Also, the quality of embryos, as indicated by the TCN, apoptotic index, and the cryosurvival rate was similar among the three groups. Our results demonstrate that despite the cryo-damages that the somatic cells could have suffered during freezing and thawing, they retained their capability of being reprogrammed by oocytes. It is possible that the genomic integrity of the cells was maintained during the freezing and thawing processes since cattle nuclei injected into enucleated oocytes have been found to develop to normal cloned embryos even after lyophilization of somatic cells, since the genome remained intact in 60% of cells [1]. Once the ability of cloned embryos produced using frozen-thawed semen-derived cells to give normal offspring is established, it could pave the way for restoration of highly valuable progeny-tested bulls that may have died years back, but have semen samples available.

At 48%, the blastocyst rate obtained in the present study, in which hand-made cloning [18] was used for SCNT (Figure S4), is higher than that of 14–34% reported in earlier studies in which micromanipulation-based SCNT was used for producing cloned embryos using cells obtained from fresh semen in cattle [6]. We obtained a blastocyst rate ranging from 34–51% for cloned embryos produced using frozen-thawed semen-derived cells. In an earlier study in cattle, no morulae or blastocysts could be obtained from cells obtained from frozen-thawed semen although cleavage occurred [6]. Besides other reasons, this failure may also be due to the use of somatic cells directly after their isolation from frozen-thawed semen without any culture and repeated subculture, as done in the present study, which is expected to eliminate the abnormal and non-mitotic cells.

Previous studies have linked developmental defects in cloned embryos to aberrant epigenetic reprogramming during early development [19]–[20]. Therefore, we studied both histone modifications (H3K9-14ac, H3K18ac, H4K5ac and H3K27me3) and DNA methylation in semen-derived somatic cells. We found that in Mu-5926, the global level of H3K27me3 was lower (P<0.05) in skin-derived cells than that in fresh and frozen thawed semen-derived cells, and a similar pattern was observed in the cloned embryos produced using these cells. No significant differences were observed in the level of H3K9/14ac among donor cells derived from skin or fresh or frozen-thawed semen or among cloned embryos produced using these three cell types. In the case of H4K5ac, the level was higher (P<0.05) in semen- than in skin-derived cells but the differences in its level among the cloned embryos produced using these cell types were not significant. The global level of DNA methylation was higher (P<0.05) in semen-derived cells than that in skin-derived cells. DNA methylation is a widely accepted gene expression silencing mark and is considered as being coupled to H3K27me3 through enzymatic interaction [21]. We found that semen- and skin-derived cells differed epigenetically. To find out the possible causes for this difference, we analyzed the relative expression levels of *HDAC1*, *DNMT1*, *DNMT3a*, *P53* and *CASPASE3* in donor cells and cloned embryos. A relationship was observed between the relative mRNA abundance of *HDAC1*, *DNMT1* and *P53* genes in the donor cells and in cloned embryos produced using these cells. The expression level of *HDAC1*, *DNMT1* and *P53* was higher (P<0.05) in the donor cells obtained from fresh or frozen-thawed semen than that in skin-derived cells. A similar pattern was observed in cloned embryos produced using these cells. However, for *CASPASE 3*, the expression level was similar among the cloned embryos produced using the three cell types although its expression level was higher (P<0.05) in the cells obtained from fresh or frozen-thawed semen than that in skin-derived cells. The relative mRNA abundance of *DNMT3a* was similar among the three cell types and among cloned embryos produced using these cells.

The ultimate test of the cloning potential of any donor cell type is its ability to produce live offspring. For examining the in vivo developmental potential of the embryos produced from both fresh and frozen-thawed semen-derived somatic cells, we transferred day 7/8 blastocysts, 2 each to 12 and 10 recipients, respectively, which resulted in one pregnancy from cloned embryos produced from fresh semen-derived somatic cells leading to the birth of an offspring weighing 55 kg through normal parturition. The calf is now 6 months of age and is normal. In case of cloned embryos produced from frozen thawed semen-derived somatic cells, two recipients were found to be pregnant, one pregnancy aborted in the first trimester and one calf born severely underweight (17 kg), which died after 12 h of birth. For parentage testing of the calf, the genomic DNA isolated from the blood of the calves, the recipients and the semen of bulls and from the cultured cells was subjected to microsatellite analysis using 13–15 buffalo-specific microsatellite markers. The genotype of the cloned calves was identical with that of the bulls and its semen-derived somatic cells but different from that of the recipient mother. These results confirm the ability of the semen-derived cells to be reprogrammed just as the conventionally used skin-derived cells despite their epigenetic differences. This opens many possibilities since a vast collection of cryopreserved semen from domestic and nondomestic species is available in cryobanks worldwide. Not only could highly precious high genetic merit progeny-tested bulls be restored, which could have immense economic importance, this technology could also be used for restoration of non-domestic animal species.

In conclusion, the results of this study demonstrate that somatic cells can be isolated from frozen-thawed or fresh semen, established in culture, and used successfully for producing live offspring through cloning. The ability of cells derived from fresh and frozen-thawed semen to produce live offspring confirms the ability of these cells to be reprogrammed. Our findings can pave the way for restoration of highly precious progeny-tested bulls, which can have immense economic importance, and can also be used for restoration of endangered species.

## Supporting Information

Figure S1 **Isolation of somatic cells from semen.** (a) Contents obtained from different fractions after percoll gradient centrifugation of semen and (b) morphological appearance of cells at different days of culture.
(TIF)

Figure S2 **Characterization and growth characteristics of somatic cells.** (a) Morphological appearance; (b) expression of cell-specific markers; (c) population doubling time; effect of attachment factors on the proliferation rate of cells derived from (d) skin and (e) frozen-thawed semen; (f) diameter and (g) viability of skin- and semen-derived somatic cells. Values with different superscripts within c, e and f differ significantly (P<0.05).
(TIF)

Figure S3 **Mean pixel intensity of H3K9/14ac, H4K5ac, H3K18ac and H3K27me3 examined by immunofluorescence staining in cells derived from skin or fresh or frozen-thawed semen.**
(TIF)

Figure S4 **Steps for production of cloned embryos using handmade cloning.** a, b, c, d, e, f & g represent immature buffalo oocytes with cumulus mass, oocytes with cumulus expansion after 21 h of maturation in vitro, making cumulus free oocytes, zona free oocytes after pronase digestion (arrow showing protrusion cone), H-33342 staining of zona free oocytes indicates that metaphases location is just behind of protrusion cone (arrow), manual enucleation on basis of protrusion cone and confirmation of enucleation by H-33342 staining respectively. h, i, j & k represent donor cells at growing culture, making single cell suspension by trypsinization, attachment of single trypsinized somatic cell with enucleated oocyte (arrow) and fused oocytes post electrofusion respectively. Arrow indicates somatic cell position after electrofusion of oocytes. l, m, n & o represent 4 cell stage cloned embryos at day 2, 8 cell stage cloned embryos at day 3, initiation of compaction stage at day 5 and group of cloned blastocysts at day 8 respectively.
(TIF)

Table S1 **Real-time PCR primers for each target gene.**
(DOCX)

Table S2 **DNA microsatellite-based origin conformity of frozen thawed-semen-derived somatic cells.**
(DOCX)

Table S3 **Parentage identity of cloned calf produced from transfer of fresh semen-somatic cells derived cloned embryos on the basis of 15 microsatellite markers.**
(DOCX)

Table S4 **Parentage identity of cloned calf produced from transfer of frozen thawed semen-somatic cells derived cloned embryos on the basis of 13 microsatellite markers.**
(DOCX)
