# Supplementary figures and images for: Hope for Restoration of Dead Valuable Bulls through Cloning Using Donor Somatic Cells Isolated from Cryopreserved Semen
Source: PLoS One. 2014 Mar 10;9(3):e90755. doi: 10.1371/journal.pone.0090755 (PMC3948694; doi:10.1371/journal.pone.0090755)

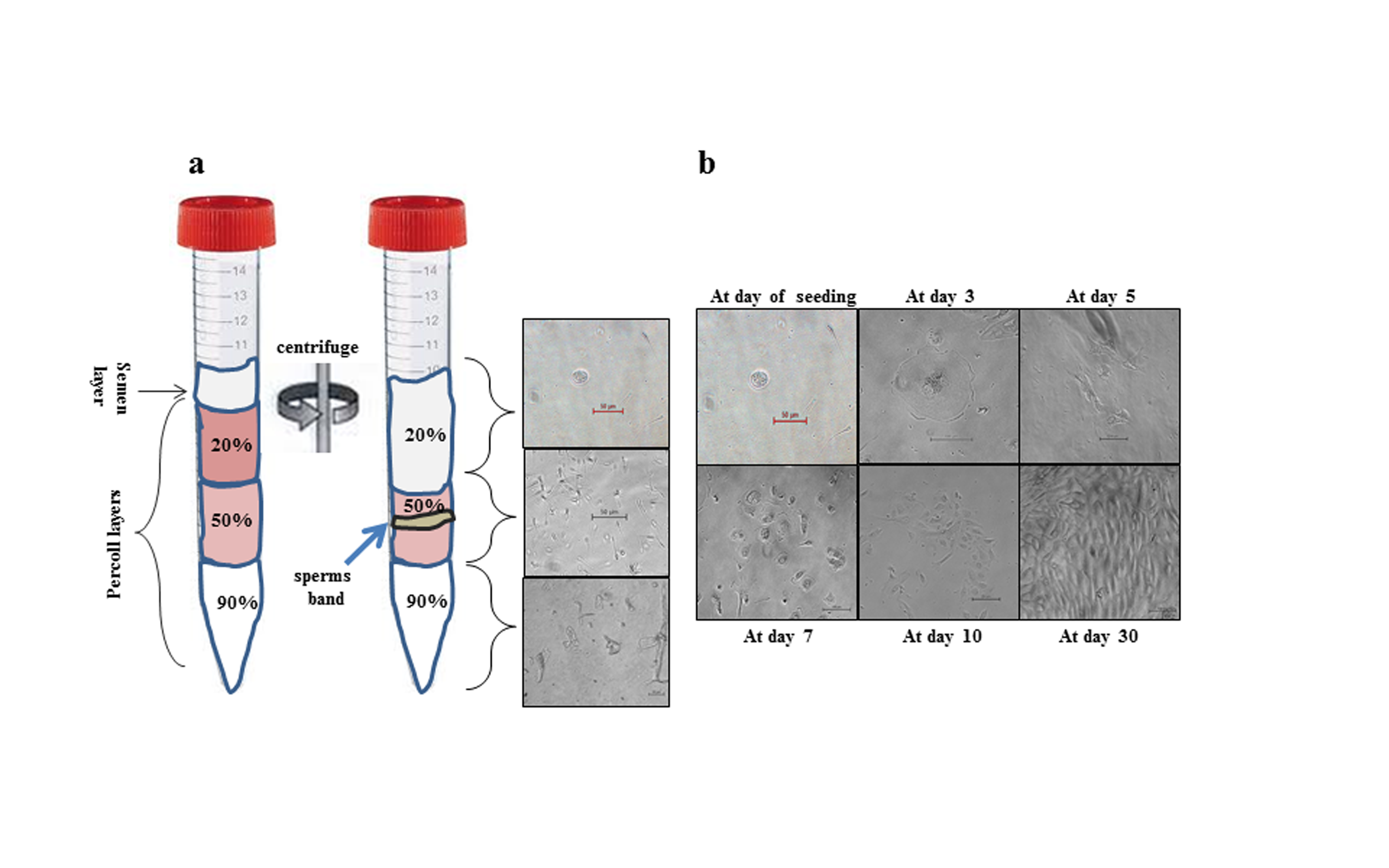

Supplement: Figure S1 — Isolation of somatic cells from semen. (a) Contents obtained from different fractions after percoll gradient centrifugation of semen and (b) morphological appearance of cells at different days of culture. (TIF) [file pone.0090755.s001.tif]

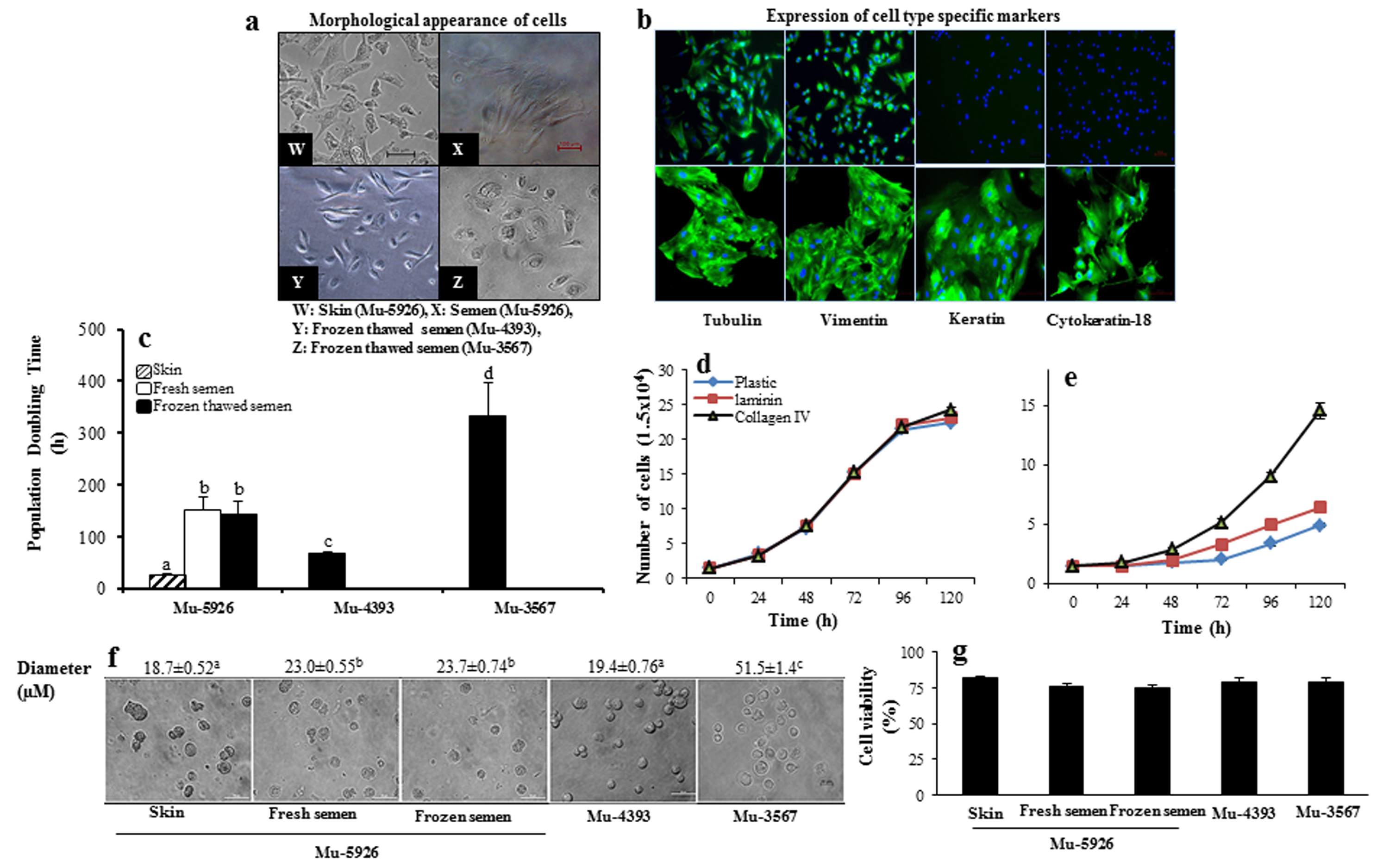

Supplement: Figure S2 — Characterization and growth characteristics of somatic cells. (a) Morphological appearance; (b) expression of cell-specific markers; (c) population doubling time; effect of attachment factors on the proliferation rate of cells derived from (d) skin and (e) frozen-thawed semen; (f) diameter and (g) viability of skin- and semen-derived somatic cells. Values with different superscripts within c, e and f differ significantly (P<0.05). (TIF) [file pone.0090755.s002.tif]

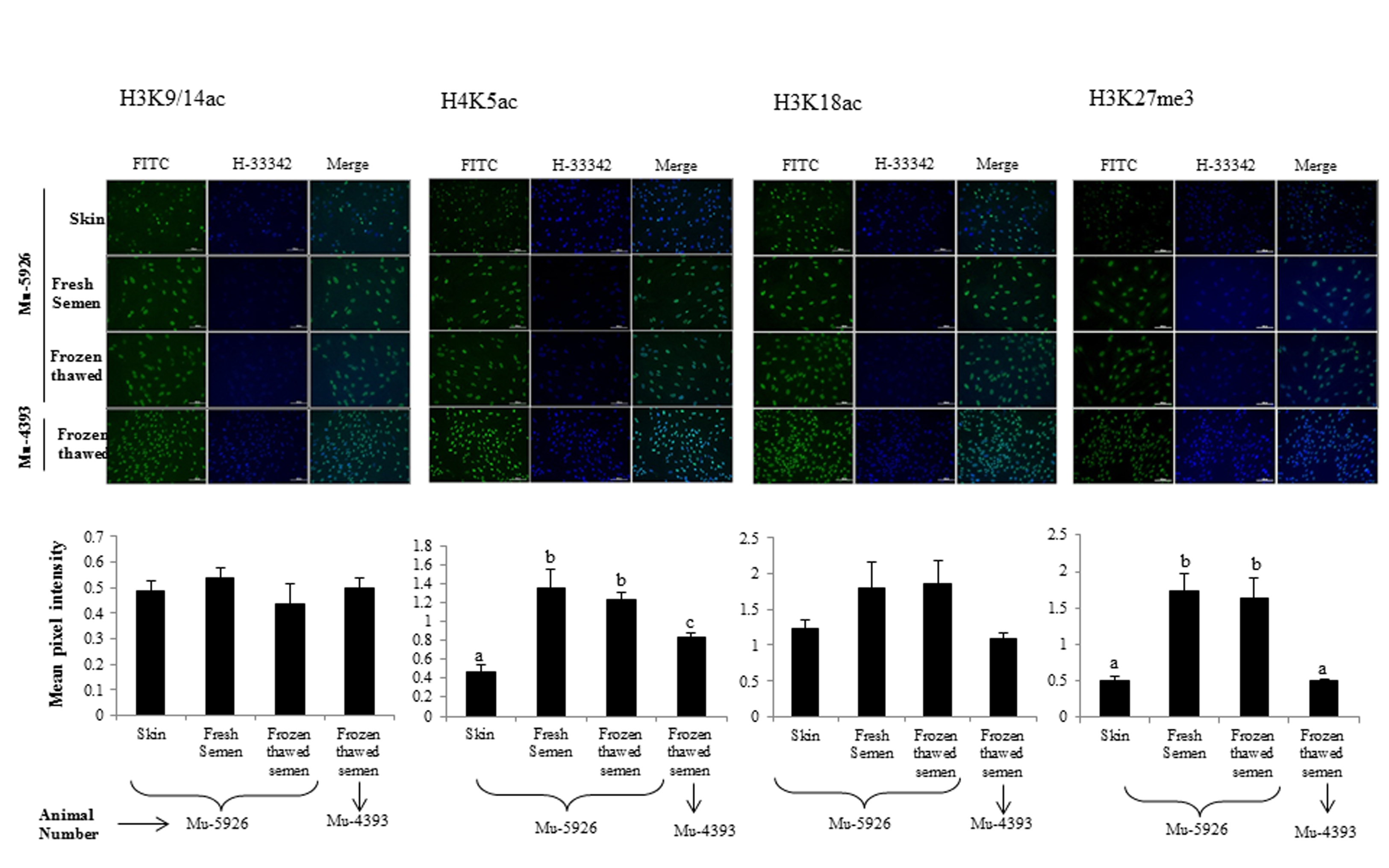

Supplement: Figure S3 — Mean pixel intensity of H3K9/14ac, H4K5ac, H3K18ac and H3K27me3 examined by immunofluorescence staining in cells derived from skin or fresh or frozen-thawed semen. (TIF) [file pone.0090755.s003.tif]

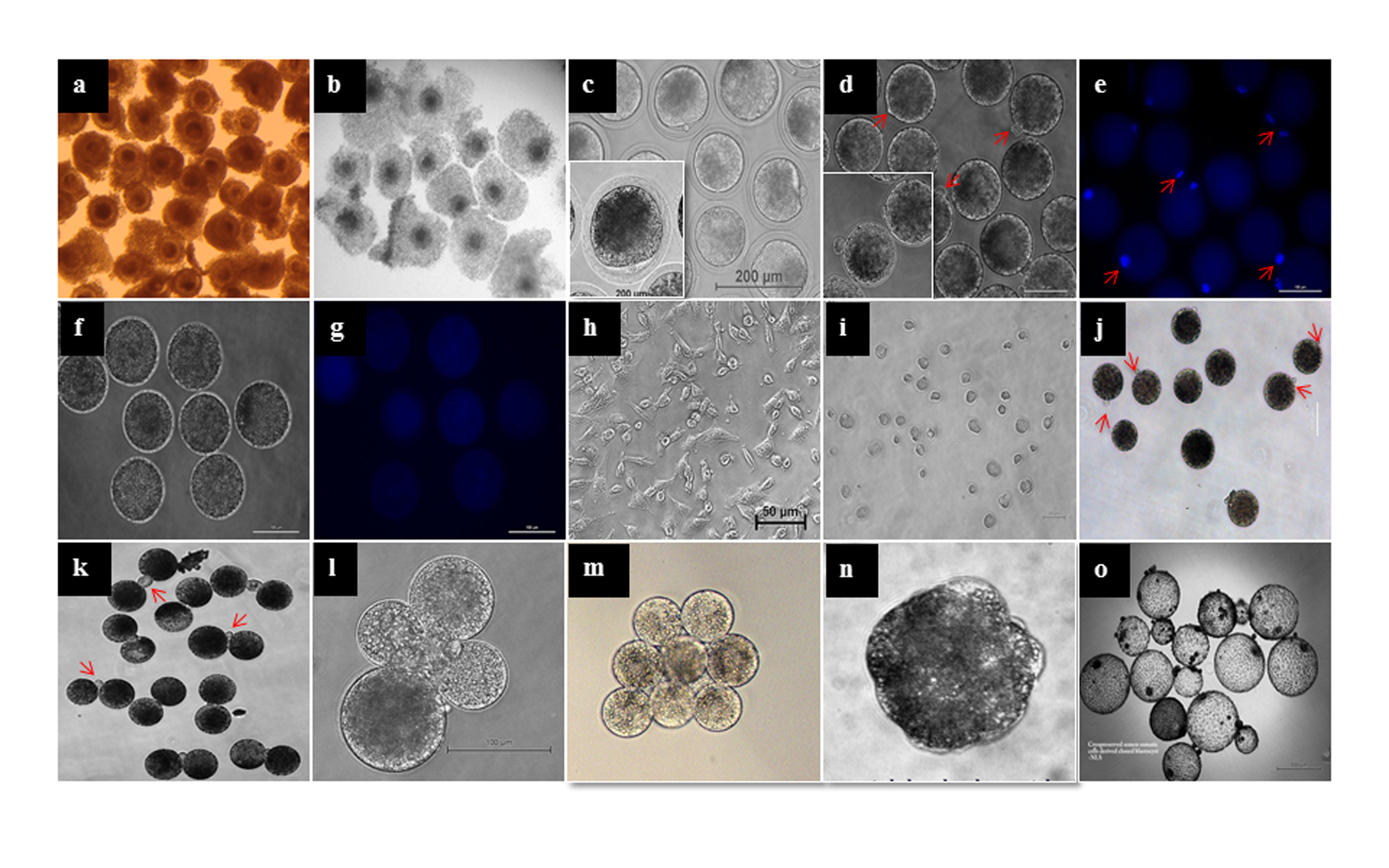

Supplement: Figure S4 — Steps for production of cloned embryos using handmade cloning. a, b, c, d, e, f & g represent immature buffalo oocytes with cumulus mass, oocytes with cumulus expansion after 21 h of maturation in vitro, making cumulus free oocytes, zona free oocytes after pronase digestion (arrow showing protrusion cone), H-33342 staining of zona free oocytes indicates that metaphases location is just behind of protrusion cone (arrow), manual enucleation on basis of protrusion cone and confirmation of enucleation by H-33342 staining respectively. h, i, j & k represent donor cells at growing culture, making single cell suspension by trypsinization, attachment of single trypsinized somatic cell with enucleated oocyte (arrow) and fused oocytes post electrofusion respectively. Arrow indicates somatic cell position after electrofusion of oocytes. l, m, n & o represent 4 cell stage cloned embryos at day 2, 8 cell stage cloned embryos at day 3, initiation of compaction stage at day 5 and group of cloned blastocysts at day 8 respectively. (TIF) [file pone.0090755.s004.tif]
