# Supplementary material for: Hope for Restoration of Dead Valuable Bulls through Cloning Using Donor Somatic Cells Isolated from Cryopreserved Semen
Source: PLoS One. 2014 Mar 10;9(3):e90755. doi: 10.1371/journal.pone.0090755 (PMC3948694; doi:10.1371/journal.pone.0090755)
Supplement: Table S1 — Real-time PCR primers for each target gene. (DOCX) [file pone.0090755.s005.docx]

Table S1: Real-time PCR primers for each target gene.

| **Gene** | **Sequence** | **Annealing temperature** | **Product size** | **Acc. No** |
| --- | --- | --- | --- | --- |
| *HDAC1* | F-ATCGGTTAGGTTGCTTCAATCTG  R- GTTGTATGGAAGCTCATTAGGGA | 58˚C | 168 | BT030718.1 |
| *DNMT1* | F-CTCAGAAGGGAGATGTGGAG  R-TAGTAGTCACAGTAGCTGAGGA | 58˚C | 138 | NM_182651.2 |
| *DNMT3a* | F-GTGCTGTCTCTATTCGATGG  R-CCATTCCTGGATATGCTTCTG | 58˚C | 188 | NM_001206502.1 |
| *P53* | F-GGAAGAATCACAGGCAGAACTC  R- ACTTCATTCGGACATTCATCCA | 58˚C | 176 | AB571118.1 |
| *CASPASE3* | F- TGGTATTGAGACAGACAGTGG  R-AGCATCTCACAAAGAAGCCTG | 58˚C | 158 | NM_001077840.1 |
| *β-ACTIN* | F-ACCACACCTTCTACAACGAG  R-GAACATGATCTGGGTCATCTTC | 58˚C | 112 | NM_001206502.1 |
