# Supplementary material for: Hope for Restoration of Dead Valuable Bulls through Cloning Using Donor Somatic Cells Isolated from Cryopreserved Semen
Source: PLoS One. 2014 Mar 10;9(3):e90755. doi: 10.1371/journal.pone.0090755 (PMC3948694; doi:10.1371/journal.pone.0090755)
Supplement: Table S2 — DNA microsatellite-based origin conformity of frozen thawed-semen-derived somatic cells. (DOCX) [file pone.0090755.s006.docx]

Table S2: DNA microsatellite-based origin conformity of frozen thawed-semen-derived somatic cells

|  | Mu-5926 | | Mu-4393 | | Mu-3567 | |
| --- | --- | --- | --- | --- | --- | --- |
| Sr. | Culture Cells | Semen Straws | Culture Cells | Semen Straws | Culture Cells | Semen Straws |
| No. |  |  |  |  |  |  |
| 1 | 150/158 | 150/158 | 158/164 | 158/164 | 154/154 | 154/154 |
| 2 | 119/119 | 119/119 | 113/120 | 113/120 | 111/119 | 111/119 |
| 3 | 270/270 | 271/271 | 259/271 | 259/271 | 261/261 | 261/261 |
| 4 | 220/220 | 220/220 | 208/220 | 208/220 | 210/210 | 210/210 |
| 5 | 129/129 | 129/129 | 126/129 | 127/129 | 119/128 | 119/128 |
| 6 | 86/86 | 86/86 | 84/86 | 84/86 | 83/88 | 83/88 |
| 7 | 255/257 | 255/257 | 255/255 | 255/255 | 257/259 | 257/259 |
| 8 | 205/214 | 205/214 | 207/222 | 207/222 | 207/209 | 207/209 |
| 9 | 209/209 | 209/209 | 209/211 | 209/211 | 209/211 | 209/211 |
| 10 | 125/130 | 126/130 | 125/128 | 126/128 | 115/134 | 115/134 |
| 11 | 88/101 | 88/101 | 88/101 | 88/101 | 88/101 | 88/101 |
| 12 | 117/120 | 117/120 | 121/133 | 121/133 | 116/120 | 116/120 |
| 13 | 82/84 | 82/84 | 82/86 | 82/86 | 78/82 | 78/82 |
