# Supplementary material for: Hope for Restoration of Dead Valuable Bulls through Cloning Using Donor Somatic Cells Isolated from Cryopreserved Semen
Source: PLoS One. 2014 Mar 10;9(3):e90755. doi: 10.1371/journal.pone.0090755 (PMC3948694; doi:10.1371/journal.pone.0090755)
Supplement: Table S3 — Parentage identity of cloned calf produced from transfer of fresh semen-somatic cells derived cloned embryos on the basis of 15 microsatellite markers. (DOCX) [file pone.0090755.s007.docx]

Table S3: Parentage identity of cloned calf produced from transfer of fresh semen-somatic cells derived cloned embryos on the basis of 15 microsatellite markers.

| **Sr. No** | **Cloned calf** | **Donor blood** | **Culture cells** | **Recipient buffalo** |
| --- | --- | --- | --- | --- |
|  | 125/125 | 125/125 | 125/125 | 125/125 |
|  | 178/178 | 178/178 | 178/178 | 178/180 |
|  | 135/135 | 135/135 | 135/135 | 135/135 |
|  | 181/187 | 181/187 | 181/187 | 181/187 |
|  | 262/267 | 262/267 | 262/267 | 262/267 |
|  | 96/96 | 96/96 | 96/96 | 96/96 |
|  | 110/110 | 110/110 | 110/110 | 110/110 |
|  | 76/96 | 76/96 | 76/96 | 76/96 |
|  | 116/116 | 116/116 | 116/116 | 116/116 |
|  | 81/96 | 81/96 | 81/96 | 81/81 |
|  | 76/76 | 76/76 | 76/76 | 76/76 |
|  | 220/249 | 220/249 | 220/249 | 220/249 |
|  | 276/302 | 276/302 | 276/302 | 276/296 |
|  | 245/247 | 245/247 | 245/247 | 241/247 |
|  | 106/106 | 106/106 | 106/106 | 108/108 |
