# Supplementary material for: Hope for Restoration of Dead Valuable Bulls through Cloning Using Donor Somatic Cells Isolated from Cryopreserved Semen
Source: PLoS One. 2014 Mar 10;9(3):e90755. doi: 10.1371/journal.pone.0090755 (PMC3948694; doi:10.1371/journal.pone.0090755)
Supplement: Table S4 — Parentage identity of cloned calf produced from transfer of frozen thawed semen-somatic cells derived cloned embryos on the basis of 13 microsatellite markers. (DOCX) [file pone.0090755.s008.docx]

| **Sr. No** | **Cloned calf** | **Donor semen straws** | **Culture cells** | **Recipient buffalo** |
| --- | --- | --- | --- | --- |
|  | 158/164 | 158/164 | 158/164 | 154/158 |
|  | 113/120 | 113/120 | 113/120 | 113/119 |
|  | 259/271 | 259/271 | 259/271 | 260/260 |
|  | 208/220 | 208/220 | 208/220 | 208/208 |
|  | 127/129 | 127/129 | 127/129 | 127/129 |
|  | 84/86 | 84/86 | 84/86 | 84/86 |
|  | 255/255 | 255/255 | 255/255 | 252/255 |
|  | 207/222 | 207/222 | 207/222 | 207/222 |
|  | 209/211 | 209/211 | 209/211 | 209/211 |
|  | 126/128 | 126/128 | 126/128 | 126/128 |
|  | 88/101 | 88/101 | 88/101 | 110/105 |
|  | 121/133 | 121/133 | 121/133 | 121/136 |
|  | 82/86 | 82/86 | 82/86 | 80/82 |

Table S4: Parentage identity of cloned calf produced from transfer of frozen thawed semen- somatic cells derived cloned embryos on the basis of 13 microsatellite markers.
